# OR7-006 – Autophagy as a player in inflammation in TRAPS

**DOI:** 10.1186/1546-0096-11-S1-A107

**Published:** 2013-11-08

**Authors:** T Bachetti, S Chiesa, P Castagnola, D Bani, E Di Zanni, A Omenetti, A D'Osualdo, A Fraldi, A Ballabio, R Ravazzolo, A Martini, M Gattorno, I Ceccherini

**Affiliations:** 1 U.O.C. Genetica Medica, Genoa, Italy; 2 Lab Rheumatology, Istituto Giannina Gaslini, Genoa, Italy; 3 San Martino, Genoa, Italy; 4 4Dept. Anatomy, Histology & Forensic Medicine, Università di Firenze, Florence, Italy; 5 Sanford-Burnham Medical Research Institute, La Jolla, CA, USA; 6 TIGEM, Naples, Italy; 7 Federico II University, Naples, Italy; 8 Molecular and Human Genetics, Baylor College of Medicine, Houston, TX, USA; 9 Dan Duncan Neurological Research Institute, Texas Children Hospital, Houston, TX, USA; 10 Pediatrics II, University of Genoa, Italy; 11 Pediatrics II, Istituto Giannina Gaslini, Genoa, Italy

## Introduction

Tumor Necrosis Factor Receptor (TNFR) Associated Periodic Syndrome (TRAPS) is a dominant autoinflammatory disorder caused by heterozygous mutations in *TNFRSF1A*, the gene encoding the TNFalpha receptor 1 (TNFR1). *TNFRSF1A* mutations induce aberrant localization and accumulation in aggregates of the mutant TNFR1 proteins, elevated levels of reactive oxygen species (ROS) and excessive inflammatory response. In accordance to the emerging role of autophagy in inflammatory response, we have recently demonstrated that mutant TNFR1 accumulation is due to a defective autophagy function, the main cellular mechanism involved in the elimination of cellular inclusions containing mutant proteins.

## Objectives

Investigation of the role of autophagy in TRAPS and search for drugs able to counteract mutant TNFR1mutant accumulation by autophagy induction.

## Methods

To search a link between *TNFRSF1A* mutations and inflammation in TRAPS, by means of both *in vitro* and *ex vivo* systems, represented by HEK293T cells transfected with expression constructs for WT and mutant TNFR1 proteins and by monocytes, derived by TRAPS patients, respectively, we have investigated the cellular response to mutant TNFR1 proteins in terms of autophagy efficiency, NF-kB activity and mutant TNFR1 localization after drugs treatments.

## Results

We have found that autophagy is the main mechanism involved in mutant TNFR1 elimination and that it is impaired in the presence of misfolded proteins, thus likely accounting for their accumulation. This compellingly accounts for TRAPS associated induction of NF-kB activity, as well as excessive IL-1b secretion and chronic inflammation.

We also show that autophagy inhibition due to TNFR1 mutant proteins can be reverted, as demonstrated by the effects of the antibiotic geldanamycin found to rescue membrane localization of mutant TNFR1 proteins, to reduce their aggregation and to counteract the enhanced inflammation by decreasing IL-1b secretion.

## Conclusion

Overall, these observations provide a rationale to the apparent paradox that so far the most effective therapy in TRAPS is represented by inhibition of the cascade signaling induced by IL-1b rather than by the use of drugs counteracting the TNFR1-mediated pathway; therefore, we propose autophagy as a novel therapeutic target for TRAPS and other inflammatory diseases.

## Disclosure of interest

None declared.

